# Quantum machine learning: a classical perspective

**DOI:** 10.1098/rspa.2017.0551

**Published:** 2018-01-17

**Authors:** Carlo Ciliberto, Mark Herbster, Alessandro Davide Ialongo, Massimiliano Pontil, Andrea Rocchetto, Simone Severini, Leonard Wossnig

**Affiliations:** 1 Department of Computer Science, University College London, London, UK; 2 Department of Engineering, University of Cambridge, Cambridge, UK; 3 Max Planck Institute for Intelligent Systems, Tübingen, Germany; 4 Computational Statistics and Machine Learning, Istituto Italiano di Tecnologia, Genoa, Italy; 5 Department of Materials, University of Oxford, Oxford, UK; 6 Institute of Natural Sciences, Shanghai Jiao Tong University, Shanghai, People’s Republic of China; 7 Theoretische Physik, ETH Zürich, Zurich, Switzerland

**Keywords:** quantum, machine learning, quantum computing

## Abstract

Recently, increased computational power and data availability, as well as algorithmic advances, have led machine learning (ML) techniques to impressive results in regression, classification, data generation and reinforcement learning tasks. Despite these successes, the proximity to the physical limits of chip fabrication alongside the increasing size of datasets is motivating a growing number of researchers to explore the possibility of harnessing the power of quantum computation to speed up classical ML algorithms. Here we review the literature in quantum ML and discuss perspectives for a mixed readership of classical ML and quantum computation experts. Particular emphasis will be placed on clarifying the limitations of quantum algorithms, how they compare with their best classical counterparts and why quantum resources are expected to provide advantages for learning problems. Learning in the presence of noise and certain computationally hard problems in ML are identified as promising directions for the field. Practical questions, such as how to upload classical data into quantum form, will also be addressed.

## 1. Introduction

In the last 20 years, owing to increased computational power and the availability of vast amounts of data, *machine learning* (ML) algorithms have achieved remarkable successes in tasks ranging from computer vision [1] to playing complex games such as Go [2]. However, this revolution is beginning to face increasing challenges. With the size of datasets constantly growing and Moore’s law coming to an end [3], we might soon reach a point where the current computational tools will no longer be sufficient. Although tailored hardware architectures, like graphics processing units (GPUs) and tensor processing units (TPUs), can significantly improve performance, they might not offer a structural solution to the problem.

Quantum computation is a computational paradigm based on the laws of quantum mechanics. By carefully exploiting quantum effects such as interference or (potentially) entanglement, quantum computers can efficiently solve selected problems [4–6] that are believed to be hard for classical machines. This review covers the intersection of ML and quantum computation, also known as *quantum machine learning* (QML). The term QML has been used to denote different lines of research such as using ML techniques to analyse the output of quantum processes or the design of classical ML algorithms inspired by quantum structures. For the purpose of this review, we refer to QML solely to describe learning models that make use of quantum resources.

The goal of this review is to summarize the major advances in QML for a mixed audience of experts in ML and quantum computation and to serve as a bridge between the two communities. Most problems will be analysed under the lens of computational complexity, which is, possibly, a unifying language for both communities. We do not aim for completeness but rather discuss only the most relevant results in quantum algorithms for learning. For the interested reader there are now a number of resources covering QML in the broader sense of the term [7,8]. For an introduction to quantum algorithms, we refer to the reviews of Montanaro [9] and Bacon & Van Dam [10], while for ML to the books by Bishop [11] and Murphy [12].

Why should an ML expert be interested in quantum computation? And why are we expecting quantum computers to be useful in ML? We can offer two reasons. First, with an ever-growing amount of data, current ML systems are rapidly approaching the limits of classical computational models. In this sense, quantum algorithms offer faster solutions to process information for selected classes of problems. Second, results in quantum learning theory point, under certain assumptions, to a provable separation between classical and quantum learnability. This implies that hard classical problems might benefit significantly from the adoption of quantum-based computational paradigms. But optimism should come with a dose of scepticism. The known quantum algorithms for ML problems suffer from a number of caveats that limit their practical applicability and, to date, it is not yet possible to conclude that quantum methods will have a significant impact in ML. We will cover these caveats in detail and discuss how classical algorithms perform in the light of the same assumptions.

Quantum computation is a rapidly evolving field but the overarching question remains: when will we have a quantum computer? Although it is not within the scope of this review to present a time line for quantum computation it is worth noting that in recent years the worldwide effort to build a quantum computer has gained considerable momentum owing to the support of governments, corporations and academic institutions. It is now the consensus that general purpose quantum computation is within a 15 year time line [13].

The review is structured as follows. We start with §2 by providing some essential concepts in quantum computation for the reader with no prior knowledge of the field. In §3, we introduce the standard models of learning, their major challenges and how they can be addressed using quantum computation. Section 4 surveys results in quantum learning theory that justify why we expect quantum computation to help in selected learning problems. We proceed in §5 by discussing how to access data with a quantum computer and how these models compare with parallel architectures. We continue by presenting different computational and mathematical techniques that find widespread application in ML, and can be accelerated with a quantum computer. More specifically, we survey quantum subroutines to speed up linear algebra (§6), sampling (§7) and optimization problems (§8). For each section, we discuss the asymptotic scaling of the classical and quantum subroutine and present some learning applications. Section 9 is dedicated to quantum neural networks. Even if neural networks are not a mathematical technique on their own, they are surveyed in a dedicated section due to their prominence in modern ML. The last two sections cover two promising applications of quantum computation in ML. In §10, we consider the case of learning under noise, while in §11 we discuss computationally hard problems in ML. We conclude with an outlook section.

## 2. Essential quantum computation

Quantum computing focuses on studying the problem of storing, processing and transferring information encoded in quantum mechanical systems. This *mode* of information is consequently called *quantum information*. The book by Nielsen & Chuang [14] is a standard introduction to the field. Loosely speaking, quantum computational models propose a probabilistic version of (time) reversible computation, i.e. computation in which the output is in one-to-one correspondence with the input. According to quantum theory, physical states are mathematically represented by density matrices, which are trace-one, positive-semi-definite matrices that generalize the concept of probability distributions. The logical states used by a quantum computational model are then identified with the physical states of the quantum system that implements them. A computation is executed reversibly by applying a sequence of unitary matrices to an initialized state. A probabilistic output is obtained according to the distribution encoded by the final density matrix.

In this framework, the fundamental unit of quantum information is the state of any quantum system with two degrees of freedom distinguishable by an observer, which then coincide with the usual logical values 0 and 1. This is called *qubit* and, for our purposes, it is a vector *ψ*=*α*_0_*e*_0_+*α*_1_*e*_1_, where $\alpha_{0},\alpha_{1}\in C$, |*α*_0_|^2^+|*α*_1_|^2^=1 and, *e*_*i*_ denotes the *i*th standard basis vector. The values of a distribution on 0 and 1 are given by {|*α*_1_|^2^,|*α*_2_|^2^}. It is a basic fact that the information content of a qubit is equivalent to a single bit. Registers of multiple qubits are assembled with the use of a tensor product. Unitary matrices acting on a small number of qubits can then be interpreted as a generalization of logic gates. The induced dynamics is responsible for interference, a key ingredient of quantum computation. By exploiting interference effects, quantum computers are able to simultaneously evaluate a function on every point of its domain. Although the result of this computation is not immediately accessible to a classical observer, the possibility of using quantum dynamics to increase the probability of determining a given property of the function is promised to allow a quantum computer to solve some computational problems exponentially faster than classical devices. Although the true roots, and extents, of quantum speed-ups are still unclear, it is believed that structure, certain symmetries and non-classical correlations play an important role in the observed advantages [15,16].

In the context of the analysis of classical data, we can exploit the encoding of quantum information to efficiently represent classical probability distributions with exponentially many points. For instance, when *v*=(*v*_1_,…,*v*_2^*n*^_) is a probability vector of size 2^*n*^, we can write an *n*-qubit state (register), $\psi =\sum_{i=1}^{2^{n}}\sqrt{v_{i}}e_{i}$.

Finally, when we use the term qubits, we always refer to idealized, error-free, objects. In practice, quantum states are extremely fragile and require extensive error correction to be shielded from the effects of noise. The theory of error correction guarantees that, if the physical errors are below a certain threshold, it is possible to correct the system efficiently. The theory of error correction is reviewed by Preskill [17]. We further discuss the types of error affecting quantum systems in §10.

### (a) Comparing the performance of classical and quantum algorithms

Computational complexity studies the resources needed to perform a given computational task. One of the major goals of this theory is to classify problems according to their time complexity, which roughly corresponds to the number of elementary steps necessary to solve the problem as a function of the size of the input. The books by Papadimitrou [18] and Arora & Barak [19] provide extensive introductions.

We define *quantum speed-up* as the advantage in runtime obtained by a quantum algorithm over the classical methods for the same task. We quantify the runtime with the asymptotic scaling of the number of elementary operations used by the algorithm with respect to the size of the input. To compare the performance of algorithms, we use the computer science notation O(f(n)), indicating that the asymptotic scaling of the algorithm is upper-bounded by a function *f*(*n*) of the number *n* of parameters characterizing the problem, i.e. the size of the input. The notation $\tilde{O}(f(n))$ ignores logarithmic factors.

In computational complexity theory, it is customary to analyse algorithms with respect to the number of queries to some efficiently implementable oracles, which can be either classical or quantum. This approach to analysing algorithms is called the *query model*. In the query model, an algorithm is said to be *efficient* if it queries the oracle a polynomial number of times. Throughout this review many speed-ups are obtained in the query model. A standard oracle for QML assumes that the classical datasets can be efficiently produced in quantum superposition. The quantum random access memory (QRAM) discussed in §5 is a possible implementation of this oracle.

## 3. Setting the problem: perspectives in machine learning

The term ML refers to a variety of statistical methods to analyse data. The prototypical goal is to infer, from a finite number of observations (the training data), the future behaviour of an unknown and possibly non-deterministic process (such as the dynamics of the stock market or the activation patterns in the human brain). In the last four decades, the field of ML has grown to such an extent that even providing a brief overview of the most prominent ideas and frameworks would require a review on its own. To this end, for the purpose of this review, we mainly focus on one of the most well-established and mature areas of research, namely supervised learning from the perspective of learning theory. To the reader interested in a more satisfying overview of ML we recommend, for instance, [12].

Learning theory aims to place the problem of learning from data on solid mathematical foundations. Typical questions that one asks in this setting are: How many examples are required to learn a given function? How much computational resource is required to perform a learning task? Depending on a number of assumptions about the data access model and on the goal of learning, it is possible to define different learning models. Two prominent ones are the *probably approximately correct* (*PAC*) framework developed by Valiant [20] and the *statistical learning theory* by Vapnik [21]. Here, a learner seeks to approximate an unknown function based on a training set of input–output pairs. Examples in the training set are assumed to be drawn from an unknown probability distribution and predictions are tested on points drawn from the same distribution. PAC and statistical learning theory model the efficiency of an estimator with two quantities: the sample complexity and the time complexity. The *sample complexity* is the minimum number of examples required to learn a function up to some approximation parameters and it is directly related to the capacity of the hypotheses space and the regularity of the data distribution; the *time complexity* corresponds to the runtime of the best learning algorithm. A learning algorithm is said to be efficient if its runtime is polynomial in the dimension of the elements of the domain of the function and inverse polynomial in the error parameters.

In these settings, the goal is to find a model that fits well a set of training examples but that, more importantly, guarantees good prediction performance on new observations. This latter property, also known as *generalization capability* of the learned model, is a key aspect separating ML from the standard optimization literature. Indeed, while data fitting is often approached as an optimization problem in practice, the focus of ML is to design statistical estimators able to ‘fit’ well future examples. This question is typically addressed with so-called *regularization* techniques, which essentially limit the expressive power of the learned estimator in order to avoid overfitting the training dataset.

A variety of regularization strategies have been proposed in the literature, each adopting a different perspective on the problem (see [11,21,22] for an introduction on the main ideas). Among the most well-established approaches, it is worth mentioning those that directly impose constraints on the hypotheses class of candidate predictors (either in the form of hard constraints or as a penalty term on the model parameters, such as in Tikhonov regularization) or those that introduce the regularization effect by ‘injecting’ noise in the problem (see §10). These ideas have led to popular ML approaches currently used in practice, such as regularized least squares [23], Gaussian process (GP) regression and classification [24], logistic regression [11] and support vector machines (SVMs) [21] to name a few.

From a computational perspective, regularization-based methods leverage on optimization techniques to find a solution for the learning problem and typically consist of a sequence of standard linear algebra operations such as matrix multiplication and inversion. In particular, most classical algorithms, such as GPs or SVMs, require a number of operations comparable to that of inverting a square matrix that has size equal to the number *N* of examples in the training set. This leads, in general, to a time complexity of $O(N^{3})$ which can be improved depending on the sparsity and the conditioning of the specific optimization problem (see §6). However, as the size of modern datasets increases, the above methods are approaching the limits of their practical applicability.

Recently, alternative regularization strategies have been proposed to reduce the computational costs of learning. Instead of considering the optimization problem as a separate process from the statistical one, these methods hinge on the intuition that reducing the computational burden of the learning algorithm can be interpreted as a form of regularization on its own. For instance, *early stopping* approaches perform only a limited number of steps of an iterative optimization algorithm (such as gradient descent) to avoid overfitting the training set. This strategy clearly entails fewer operations (fewer number of steps) but can be shown theoretically to lead to the same generalization performance of approaches such as Tikhonov regularization [22]. A different approach, also known as *divide and conquer*, is based on the idea of distributing portions of the training data onto separate machines, each solving a smaller learning problem, and then combining individual predictors into a joint one. This strategy benefits computationally from both the parallelization and reduced dimension of distributed datasets and it has been shown to achieve the same statistical guarantees of classical methods under suitable partitions of the training data [25]. A third approach that has recently received significant attention from the ML community is based on the idea of constraining the learning problem to a small set of candidate predictors, obtained by randomly sampling directions in a larger, universal hypotheses space (namely a space dense in the space of continuous function). Depending on how such sampling is performed, different methods have been proposed, the most well-known being random features [26] and Nystrom approaches [27,28]. The smaller dimensionality of the hypotheses space automatically provides an improvement in computational complexity. It has been recently shown that it is possible to obtain equivalent generalization performance to classical methods also in these settings [29].

For all these methods, training times can be typically reduced from the $O(N^{3})$ of standard approaches to $\tilde{O}(N^{2})$ while keeping the statistical performance of the learned estimator essentially unaltered.

Because the size of modern datasets is constantly increasing, time complexities of the order of $\tilde{O}(N^{2})$ might still be too demanding for practical applications. In this regard, quantum computation could offer the potential to further improve the efficiency of such methods, allowing them to scale up significantly. Indeed, through a number of quantum algorithms for linear algebra, sampling and optimization techniques, we could in principle obtain up to exponential speed-ups over classical methods. However, as will be discussed in §6, current QML methods require fast memory access and particular data structures that might limit their applicability in real settings. Nevertheless, as we will discuss in the following section, a number of results in quantum learning theory point, under specific assumptions, to a clear separation between classical and quantum learning paradigms in some specific settings.

## 4. ‘Can we do better?’: insights from quantum learning theory

Learning theorists have been interested in studying how quantum resources can affect the efficiency of a learner since the 1990s. Although different learning models have been translated into the quantum realm, here we focus on the quantum version of the PAC model. The reason for this choice is that, in this model, we have results for both the sample and the time complexity. For an extensive overview of the known results in quantum learning theory, we refer the reader to the review by Arunachalam & de Wolf [30].

The quantum PAC model was introduced in [31]. Here, it is assumed that the learner has access to a quantum computer and to an oracle that returns the training set in quantum superposition. In terms of sample complexity, it has been shown in a series of papers, which constantly improved the bounds until reaching provable optimality [32–35], that the quantum PAC model under an unknown distribution and standard PAC are equivalent up to constant factors. This implies that, in general, quantum mechanics does not help to reduce the amount of data required to perform a learning task. However, if one considers a different learning model, such as the exact learning framework developed by Angluin [36], it is possible to prove that quantum learners can be polynomially more efficient than classical learners in terms of the number of queries to the data oracle [32,37].

Although quantum and classical examples are equivalent up to constant factors when learning under general distributions, the quantum PAC model can offer advantages over its classical counterpart in terms of time complexity. One of the central problems studied in the classical literature is the learnability of disjunctive normal forms (DNFs). To date, the time complexity of the best algorithm for learning DNFs under an unknown distribution is exponential [38]. A number of assumptions can be made to relax the hardness of the problem. For instance, if the learner is provided with examples drawn from the uniform distribution, then the runtime of the best learner becomes quasi-polynomial [39]. For the sake of completeness, we note that the methods presented in §3, like SVMs, have been shown to not be able to learn efficiently DNF formulae [40,41]. The learnability of DNF formulae has also been studied in the quantum PAC model [31]. Here DNFs have been shown to be efficiently learnable under the uniform distribution. This quantum speed-up is obtained through an efficient algorithm [42] that allows exponentially faster sampling from the probability distribution described by the coefficients of a Boolean Fourier transform. Interestingly, DNF formulae can be shown to be efficiently learnable under noise. We will return to this point in §10.

Another case where it is believed that learning can be performed efficiently only when the learner has access to quantum resources is based on a class of functions developed by Kearns & Valiant [43]. This class is provably hard to learn under the assumption that factoring Blum integers is also hard (an assumption widely believed to be true; for a brief introduction to the concept of hardness in computational complexity, see §11). Servedio & Gortler [32] noted that owing to Shor’s quantum factoring algorithm [4] this class of functions can be learned efficiently in the quantum PAC model.

The results coming from the quantum learning theory literature show that by carefully exploiting quantum mechanical effects, depending on the type of learning model considered, it is possible to have a better generalization error (i.e. we can learn with fewer examples) or we can learn functions that would otherwise be hard for classical learners.

## 5. Data access, communication and parallelism

One of the roots of the speed-ups theorized in quantum computation is the ability to process information in quantum superposition [14,44]. Because ML is ultimately about analysing vast amounts of data, it is important to address the question of how data are turned into quantum superposition. We distinguish between two types of algorithms: those that operate on quantum data (i.e. data that are the output of a quantum process, for example a quantum chemistry problem) and those that seek to process data stored in a classical memory. The first case is ideal for QML. The data are ready to be analysed and we do not have to spend computational resources to convert the data into quantum form. The second case is more elaborate as it requires a procedure that encodes the classical information into a quantum state. As we will see, the computational cost of this operation is particularly relevant to determine whether we can obtain quantum speed-ups in ML for classical data.

Let us assume that one wants to process *N* *d*-dimensional classical vectors with a quantum algorithm. The *quantum random access memory* (QRAM) [45,46] is a quantum device that can encode in superposition *N* *d*-dimensional vectors into *log*(*Nd*) qubits in O(log(Nd)) time by making use of the so-called ‘bucket-brigade’ architecture. The idea is to use a tree structure where the *Nd* leaves contain the entries of the N vectors in $R^{d}$. The QRAM, with a runtime of O(log(Nd)), can return a classical vector in quantum superposition efficiently. However, the number of physical resources it requires scales as O(Nd). As we will see, this exponential scaling (with respect to the number of qubits) has been used to question whether the QRAM can be built in an experimental setting or whether it can provide a genuine computational advantage [7,47]. Fundamentally, the issue can be related to whether the exponential number of components needs to be continuously ‘active’. The proponents of the QRAM [45,46] claim that only O(log(Nd)) components need to be active, while the others can be considered as ‘non-active’ and error-free. Whether this assumption holds in an experimental setting is unclear [48]. We now proceed to discuss its implications.

The first issue that appears with QRAM is whether all the components require to be error-corrected (we briefly discuss errors in quantum computation in §10). Indeed, if the exponential physical resources required full error correction, then it would be impractical to build the device in an experimental setting. Arunachalam *et al.* [48] addressed this question and showed that, given a certain error model, algorithms that require to query the memory a polynomial number of times (like the quantum linear system algorithm presented in §6) might not require fault-tolerant components. However, for superpolynomial query algorithms, like Grover’s search [49] (a subroutine required, for example, in some of the quantum methods for training-restricted Boltzmann machines discussed in §9), the QRAM requires error-corrected components.

A second problem related to the exponential number of resources in an active memory has been raised by Aaronson [47] and by Steiger & Troyer [50]. The authors argue that the only fair comparison of a system which requires an exponential number of resources is with a parallel architecture with a similar amount of processors. In this case, many linear algebra routines, including solving linear systems and singular value decomposition, can be solved in logarithmic time [51].

A third caveat of the QRAM is the requirement of having data distributed in a relatively uniform manner over the quantum register. As pointed out in [47,52], if that was not the case, the QRAM would violate the search lower bounds proved in [53]. In the case of non-uniformly distributed data, the QRAM is no longer efficient and takes $O(\sqrt{N})$ to turn the classical dataset into quantum superposition.

As a last comment on the QRAM, the possibility of loading the data in logarithmic time, when the size of the data is considerable, can be controversial due to speed of communication arguments. In fact, as suggested in [47], latency can play a role in big memory structures. In particular, a lower bound on the distance which the information has to travel implies a lower bound on latency, due to considerations on the limits set by the speed of light. In a three-dimensional space, these are given by $O(\sqrt[{3}]{Nd})$. In practice, these considerations will only dominate if the amount of memory is extremely large but, because in QML we aim at datasets that surpass the current capability of classical computers, this bound is a potential caveat.

In conclusion, the QRAM allows data to be uploaded efficiently but might be hard to implement experimentally or might not allow a genuine quantum advantage if we take into account all the required resources. Notably, the fast data access guaranteed by the QRAM is only required for QLM algorithms that run in sublinear time. Although many known QML algorithms run in sublinear time, quantum learning theory suggests that for some classically hard problems quantum resources might give exponential advantages. In this case, a memory structure that can prepare a quantum superposition in polynomial time (i.e. in O(Nd)) can still be sufficient to maintain a quantum speed-up compared with the classical runtime. We will discuss the hard classical learning problem in §11.

Finally, we note that, although the QRAM, due to its generality, is the most widely used memory structure in QML algorithms, other protocols to encode classical data in superposition exist. For example, a technique developed by Grover & Rudolph [54] allows one to generate a quantum superposition that encodes an approximate version of a classical probability distribution provided its density is efficiently integrable.

## 6. Fast linear algebra with quantum mechanics

A significant number of methods in the QML literature are based on fast quantum algorithms for linear algebra. In this section, we present the two main quantum subroutines for linear algebra: a quantum algorithm for matrix inversion and a quantum algorithm for singular value decomposition. We summarize the major applications of these techniques to ML problems and how they compare with classical and parallel implementations in table 1.

**Table 1.** Quantum linear algebra algorithms and their ML applications. When carefully compared with classical versions that take into account the same caveats, quantum algorithms might lose their advantages. *C*, *Q* and *P* indicate, respectively, the asymptotic computational complexity for classical, quantum and parallel computation. We remind the reader that, to date, memory and bandwidth limits in the communication between processors make the implementation of certain parallel algorithms unrealistic. We remark that asymptotic scalings are only an indication of potential runtime differences and solely by benchmarking the algorithms on quantum hardware we will obtain clear insights on their performance. Given an *N*×*N*-dimensional matrix *A*, we denote by *k* the number of singular values that are computed by the algorithm, by *s* the sparsity and by *κ* the condition number. For approximation algorithms *ϵ* is an approximation parameter. In other cases, it denotes the numerical precision. Classical algorithms return the whole solution vector. Quantum algorithms return a quantum state; in order to extract the classical vector, one needs O(N) copies on the state.

problem	solving linear system of equations	singular value estimation
scaling	$C:\tilde{O}(\sqrt{\kappa }\;\hat{s}N\log\left(1/\epsilon \right))$ [55]^*a*^	$C:O(k^{2}N\log\left(1/\delta \right)/\epsilon )$ [56]^*d*^
	$Q:\tilde{O}(s^{2}\kappa^{2}\log\left(N\right)/\epsilon )$ [57]^*b*^	$Q:O(\log\left(N\right)\epsilon^{-3})$[58]
	$P:O(\log^{2}(N)\log\left(1/\epsilon \right))$ [51]^*c*^	$P:O(\log^{2}(N)\log\left(1/\epsilon \right))$ [51]^*e*^
applications	least-squares SVM [59]	recommendation systems [60]
	GP regression [61]	linear regression [62]
	Kernel least squares [62]	principal component analysis [58]^*f*^

^*a*^An approximate algorithm that can be applied to dense matrices. Here $\hat{s}N$ is the number of entries in the matrix *A* and $\hat{s}$ alludes to the number of entries per row.^*b*^Exact but does not output the solution vector and works only for sparse matrices (more details can be found in §6).^*c*^Requires $O(N^{4})$ parallel units and is numerically unstable due to high sensitivity to rounding errors. Stable algorithms such as Gaussian elimination with pivoting or parallel *QR*-decomposition require O(N) time using $O(N^{2})$ computational units [63].^*d*^An approximate algorithm which returns a rank *k*-approximation with probability 1−*δ* and has an additional error *ϵ*∥*A*∥_*F*_. Exacts methods for an *N*×*M* matrix scale with $\min\{MN^{2},NM^{2}\}$.^*e*^Calculates SVD by computing the eigenvalue decomposition of the symmetric matrix *AA*^*T*^.^*f*^Works on dense matrices that are low-rank approximable. Finally, we note that there exist efficient, classical, parallel algorithms for sparse systems, where s=O(log⁡N) [64,65]. Probabilistic numerical linear algebra also allows selected problems to be solved more efficiently and, under specific assumptions, even in linear time and with bounded error [66].

### (a) Fast matrix inversion: the quantum linear system algorithm

Solving linear systems of equations is a ubiquitous problem in ML. As discussed in §3, many learning problems, such as GPs or SVMs, require the inversion of a matrix. For a system of linear equations *Ax*=*b* with $A\in R^{N\times N}$ and $x,b\in R^{N}$, the best classical algorithm has a runtime of $O(N^{2.373})$ [67]. However, due to a large pre-factor, the algorithm is not used in practice. Standard methods, for example, based on QR-factorization take $O(N^{3})$ steps [68].

The *quantum linear system algorithm* (QLSA) [57], also known as HHL after the three authors Harrow, Hassidim and Lloyd, promises to solve the problem in $\tilde{O}(\log(N)\kappa^{2}s^{2}/\epsilon )$, where *κ* is the condition number (defined to be the ratio of the largest to the smallest eigenvalue), *s* is the sparsity or the maximum number of non-zero entries in a row and column of *A*, and *ϵ* is the precision to which the solution is approximated. The precision is defined as the distance of the solution vector $\bar{a}$ to the true result *a*, which is given by $∥\bar{a}-a∥=\sqrt{1-2\text{Re}\langle \bar{a},a\rangle }\leq \epsilon$. Ambainis [69] and Childs *et al.* [70] improved the runtime dependency of the algorithm in *κ* and *s* to linear and the dependency in *ϵ* to poly-logarithmic.

Although the QLSA solves matrix inversion in logarithmic time, a number of caveats might limit its applicability to practical problems [47]. First, the QLSA requires the matrix *A* to be sparse. Second, the classical data must be loaded in quantum superposition in logarithmic time. Third, the output of the algorithm is not *x* itself but a quantum state that encodes the entries of *x* in superposition. Fourth, the condition number must scale at most sublinearly with *N*. An interesting problem that satisfies these requirements is discussed in [71].

Recently, Wossnig *et al.* [72] addressed the first caveat. By using a quantum walk-based approach, the authors derived an algorithm that scales as $\tilde{O}(\kappa^{2}∥A{∥}_{F}\log N/\epsilon )$ and can also be applied to dense matrices (however, in this case, the speed-up is only quadratic because $∥A{∥}_{F}=\sqrt{N}$). This result has been improved in [60], currently the best known lower bound for matrices with this property.

The second caveat inherits the same issues of the QRAM discussed in §5: it is an open question whether we can practically load classical data in quantum superposition in logarithmic time.

The third caveat is a common pitfall of quantum algorithms. As pointed out by Childs [73] and Aaronson [47], in order to retrieve classical information from the quantum state, we need at least a number of measurements that are proportional to *N*. This would destroy every exponential speed-up. One way forward is to use the QLSA only to compute certain features of the classical vector, which can be extracted efficiently using quantum mechanics, for example the expected value *x*^*T*^*Ax* of a matrix *A*. A number of other possible applications are discussed in [47].

It is then natural to question how quantum algorithms compare with their classical analogues after all the caveats have been taken into account. For example, it is possible to show that calculating an expectation value of the form *x*^*T*^*Ax* can be done in time linear in the sparsity of the matrix *A*, using classical sampling methods. Furthermore, conjugated gradient descent can obtain the full solution of the linear system (also when *A* is not sparse) in linear time in the dimensionality and, in most cases, the sparsity of *A* [55]. We present a general comparison of the asymptotic scalings of classical, quantum and parallel algorithms for linear algebra and their major applications in ML in table 1.

Comparing algorithms based on their worst case running time may not be the right approach when considering their practical applicability, as is commonly done in ML. Indeed, despite its worst case running time, an algorithm solving a given problem will often terminate much faster: average-case complexity can be much lower than worst case. Furthermore, *smoothed analysis* [74,75] provides a framework for studying the time performance of an algorithm in the presence of realistic input noise distributions. This gives another way to quantify the complexity of algorithms. To date, no quantum algorithm has been analysed in the smoothed analysis framework.

Statistical considerations can also lead to interesting insights on the computational hardness of a learning problem. Kernel-regularized least squares provide a good example. Under standard technical assumptions on the target function of the learning problem, computational regularization methods for kernel-regularized least squares [22,29,76] (see §3) achieve the optimal learning rate of $\epsilon =O(N^{-1/2})$ while requiring only $\tilde{O}(N^{2})$ operations. With optimal learning rates we mean that any learning algorithm cannot achieve better prediction performance (uniformly) on the class of problems considered. Interestingly, such assumptions also allow us to derive estimates for the condition number of the kernel matrix to be of order $\kappa =O(N^{1/2})$ [77]. The corresponding quantum scaling for the inversion of the kernel matrix is $\tilde{O}(N^{2})$ and it is therefore comparable to that of computational regularization methods implementable on classical machines (which, in addition, provide the full solution vector).

Finally, it is worth comparing the QLSA with classical parallel methods for matrix inversion. In the parallel model of computation [78], inverting an *N*×*N* matrix takes $O({\text{log}}^{2}(N))$ computational steps using a number of processors which are of order poly(*N*) (a crude upper-bound of $O(N^{4})$ is given by [51]). Although the parallel model of computation does not resemble the actual behaviour of parallel machines, it can be a fair comparison considering that quantum computers might also face connectivity issues and hence communication overheads among the qubits. In particular when exponentially large amounts of error-corrected qubits are required, as with the QRAM, it is likely that latency issues arise.

To conclude, the QLSA is a logarithmic time quantum algorithm for matrix inversion, a task arising in many learning problems. However, a number of caveats that include the requirement of a logarithmic access time to the memory and the impossibility of retrieving the solution vector with one measurement lead to the question of whether classical or parallel algorithms that make use of the same assumptions obtain similar, or better, runtimes. In this respect, experimental implementations will greatly contribute to assessing the true potential of these methods in realistic scenarios.

### (b) Quantum singular value estimation

The *singular value decomposition* (SVD) of an *M*×*N*, rank *r* matrix *A* is a factorization of the form *A*=*UΣV* ^†^, where *U* and *V* are, respectively, *M*×*M* and *N*×*N* unitary matrices and *Σ* is an *M*×*N* diagonal matrix with *r* positive entries *σ*_1_,…,*σ*_*r*_ which are called the *singular values* of *A*.

Singular value estimation is a fundamental tool in many computational problems and applications ranging from matrix inversion for linear regression to matrix approximation [68]. It is also of particular interest for problems of dimensionality reduction such as *principal component analysis* (PCA) [79]. Classically, finding such a decomposition is computationally expensive, and for *M*>*N* it takes $O(MN^{2})$ [80].

Prakash and Kerenidis [52,60] introduced the *quantum singular value estimation* (QSVE) algorithm, based on Szegedy’s work on quantum walks [81], which runs in time $O(∥A{∥}_{F}\log MN/\epsilon )$. Their algorithm returns an estimate of the singular values ${\tilde{\sigma }}_{i}$ such that $|\sigma_{i}-{\tilde{\sigma }}_{i}|\leq \epsilon$. As for the QLSA, the QSVE algorithm outputs the singular values in quantum superposition. As such, in order to read out all the *r*-values, the algorithm must be run O(Nlog⁡N) times, thus destroying any exponential speed-up. However, it is still possible to construct useful applications of the QSVE algorithm. For example, Kerenidis & Prakash [60] proposed a recommendation system which runs in O(poly(r)polylog⁡(MN)) (assuming a good *r*-rank approximation of the preference matrix).

We note that the QSVE algorithm requires an oracle that can prepare quantum states that encode the rows and the columns of the matrix *A* in poly-logarithmic time. It is possible to implement this oracle with the QRAM, and hence it will inherit the caveats discussed in §5.

An alternative method for QSVE has been proposed by Lloyd *et al.* [58]. The scaling of this algorithm is quadratically worse in *ϵ* but the requirements on the memory structure are less stringent than in [60]. This is advantageous in some applications, like analysing the principal components of kernel matrices [59].

## 7. Quantum methods for sampling

Many learning problems of practical interest, for example exact inference in graphical models, are intractable with exact methods. We discuss in detail hard learning problems in §11. Sampling methods are a common technique to compute approximations to these intractable quantities [82]. There is a rich literature on sampling methods [83–87]. The most commonly used ones are Monte Carlo methods and in particular the *Markov chain Monte Carlo* (MCMC). The quantum algorithms discussed in this section are devoted to speed up MCMC methods.

MCMC methods [88], like Gibbs sampling or the Metropolis algorithm, allow sampling from a probability distribution *Π* defined over a state space using a Markov chain that after a number of steps converges to the desired distribution (in practice one will only reach a distribution which is *ϵ*-close). The number of steps *τ* required to converge to *Π* is referred to as the *mixing time*. Estimating the mixing time can be reduced to bounding the spectral gap *δ*, which is the distance between the largest and the second largest eigenvalue of a stochastic map that evolves the Markov chain. The mixing time satisfies the inequality $\tau \geq (1/2\delta )\log{\left(2\epsilon \right)}^{-1}$ and it is possible to show [88,89] that, for the classical MCMC algorithm, *τ* is of the order of $O(1/(\delta \log\left(1/\Pi^{*}\right)))$, where Π* is the minimum value of *Π*.

Recently, there has been a significant interest in quantum algorithms that allow speeding up of the simulations of the stochastic processes used in MCMC. A common feature of these algorithms is a quadratic speed-up in terms of spectral gap, inverse temperature, desired precision or the hitting time. Advances in this field include algorithms for thermal Gibbs state preparation [90–96] which provide polynomial speed-ups in various parameters, such as the spectral gap. Other methods have introduced the concept of quantum hitting time of a quantum walk [81,96–100]. In this framework, it is possible to obtain a polynomial speed-up with respect to most classical variants (this can be exponential for the hitting time). A number of other algorithms accelerate classical Monte Carlo methods applied to the estimation of quantities such as expectation values and partition functions, which play a major role in physics [9,96,101].

## 8. Quantum optimization

As discussed in §3, optimization methods are a fundamental building block of many ML algorithms. Quantum computation provides tools to solve two broad classes of optimization problems: semi-definite programming and constraint satisfaction problems.

### (a) Quantum algorithms for semi-definite programming

Semi-definite programming [102,103] is a framework for solving certain types of convex optimization problems. Semi-definite programs (SDPs) find widespread applications in ML [104–106]. In an SDP, the objective is to minimize a linear function of an *N*×*N* positive-semi-definite matrix *X* over an affine space defined by a set of *m* constraints. The best known classical SDP solvers [107] run in time $O(m(m^{2}+n^{\omega }+mns)\log^{O(1)}(mnR/\epsilon ))$, where *ϵ* is an approximation parameter, *ω*∈[2,2.373) is the optimal exponent for matrix multiplication, *s* is the sparsity of *A* and *R* is a bound on the trace of an optimal *X*.

Based on a classical algorithm to solve SDPs by Arora & Kale [108], which has a runtime of $\tilde{O}(nms{\left(Rr/\epsilon \right)}^{4}+ns{\left(Rr/\epsilon \right)}^{7})$, where *r* is an upper bound on the sum of the entries of the optimal solution to the dual problem, in 2016 Brandão & Svore [109] developed a quantum algorithm for SDPs that is quadratically faster in *m* and *n*. The dependence on the error parameters of this result has been improved in [110]. In this work, the authors obtain a final scaling of $\tilde{O}\left(\sqrt{mn}s^{2}{\left(Rr/\epsilon \right)}^{8}\right)$.

The main problem of these quantum algorithms is that the dependence on *R*,*r*,*s* and 1/*ϵ* is considerably worse than in [108]. This quantum algorithm thus provides a speed-up only in situations where *R*,*r*,*s*,1/*ϵ* are fairly small compared with *mn* and, to date, it is unclear whether there are interesting examples of SDPs with these features (for more details, see [110]).

### (b) Quantum algorithms for constraint satisfaction problems

In a *constraint satisfaction problem* (CSP), we are given a set of variables, a collection of constraints, and a list of possible assignments to each variable [111]. The task is to find values of the variables that satisfy every constraint. This setting prompts exact and approximate cases. For many families of CSPs efficient algorithms are unlikely to exist. Two quantum algorithms are known for CSPs: the quantum approximate optimization algorithm and the quantum adiabatic algorithm (QAA). Owing to its generality and a profoundly different way of exploiting quantum evolution, the latter algorithm is also regarded as an independent computational model called *adiabatic quantum computation* (AQC). We will provide a brief introduction to AQC in the following paragraphs.

#### (i) The quantum approximate optimization algorithm

The *quantum approximate optimization algorithm* (QAOA), developed in 2014 by Farhi, Goldstone and Gutman, is a quantum method to approximate CSPs [112]. The algorithm depends on an integer parameter *p*≥1 and the approximation improves as *p* increases. For small values of *p*, the QAOA can be implemented on a shallow circuit. As argued in [113], this feature makes the QAOA a good candidate for first-generation quantum hardware.

For certain combinatorial optimization problems, the QAOA can give approximation ratios that are better than what can be achieved by random sampling [112] but worse than the best classical solvers. In specific instances of MAX-*k*XOR the QAOA with *p*=1 was believed to outperform the best classical solver [114]. This sparked further research in the classical community and Barak *et al.* [115] designed a classical algorithm able to outperform the quantum scaling.

#### (ii) The quantum adiabatic algorithm

The QAA [116] is an optimization method that operates in the adiabatic model of quantum computation. The QAA can be thought of as a quantum analogue of simulated annealing [117]. The algorithm encodes the solution to a computational problem in the unknown ground state of a quantum system (usually an Ising spin glass Hamiltonian). By starting off in the ground state of a known and easy to implement Hamiltonian, the QAA exploits a slow, time-dependent Hamiltonian dynamics to obtain the solution to the problem. If the evolution is slow enough, the quantum adiabatic theorem [118] guarantees that the system will reach the desired ground state. If the energy barriers have specific configurations (e.g. tall and narrow) and the energy gap between the ground state and the first excited state remains large enough, the algorithm can obtain significant speed-ups over classical simulated annealing [119,120].

Although QAA and AQC are usually considered synonyms in the literature, we shall keep the two concepts distinct to mark the difference between the computational model and the algorithm. Another name which is frequently used in the literature as a synonym of QAA and AQC is *quantum annealing* (QA). Although there is not a clear consensus in the literature over the differences between these three concepts, we refer to QA only when the adiabatic evolution occurs at non-zero temperature.

Aharonov *et al.* [121] showed that AQC is universal for quantum computation, i.e. it is capable of solving any computational problem that can be solved by a quantum computer. Although it is clearly possible to encode NP-hard problems [122], quantum mechanics is not expected to solve these in polynomial time (however, the scaling constants of the quantum algorithm might be smaller). Finally, it is important to note that the adiabatic algorithm lacks worst case upper bounds on its runtime. Its performance has been analysed with numerical experiments [123–128]. However, these are limited to small-size systems and only running the algorithm on actual hardware will be able to determine the strength of this approach.

## 9. Quantum neural networks

The term *artificial neural network* (ANN) denotes a variety of models which have been widely applied in classification, regression, compression, generative modelling and statistical inference. Their unifying characteristic is the alternation of linear operations with, usually preselected, nonlinear transformations (e.g. sigmoid functions) in a potentially hierarchical fashion.

While in the last decade neural networks have proved successful in many applications, fundamental questions concerning their success remain largely unanswered. Are there any formal guarantees concerning their optimization and the predictions they return? How do they achieve good generalization performance despite the capacity to completely overfit the training data?

ANNs have been extensively studied in the QML literature. The major research trends have focused on accelerating the training of classical models and on the development of networks where all the constituent elements, from single neurons to training algorithms, are executed on a quantum computer (a so-called *quantum neural network*). The first works on quantum neural networks appeared in the 1990s [129] and a number of papers have been published on the topic. However, it is worth noticing that the field has not reached a level of scientific maturity comparable to the other areas of QML discussed in this review. Possible reasons for the difficulties encountered in making progress in this area can be traced to the inherent differences between the linearity of quantum mechanics and the critical role played by nonlinear elements in ANNs or the fast developments occurring in the field of classical ANNs.

The literature on accelerated training of neural networks using quantum resources has mainly focused on *restricted Boltzmann machines* (RBMs). RBMs [130] are generative models (i.e. models that allow new observational data to be generated based on prior observations) that are particularly apt to be studied from a quantum perspective due to their strong connections with the Ising model. It has been shown that computing the log-likelihood and sampling from an RBM is computationally hard [131]. MCMC methods are the standard techniques used to overcome these difficulties. Nonetheless, even with MCMC the cost of drawing samples can be high [132] for models with several neurons. Quantum resources can help to reduce the training cost.

There are two main classes of quantum techniques to train RBMs. The first one is based on methods from quantum linear algebra (discussed in §6) and quantum sampling (discussed in §7). Wiebe *et al.* [133] developed two algorithms to efficiently train an RBM based on amplitude amplification [134] and quantum Gibbs sampling. These obtain a quadratic improvement in the number of examples required to train the RBM, but the scaling of the algorithm is quadratically worse in the number of edges than in contrastive divergence [135]. A further advantage of the approach proposed in [133] is that it can be used to train full Boltzmann machines (a classical version of this algorithm has also been proposed [136]). A full Boltzmann machine is a type of Boltzmann machine where the neurons correspond to the nodes of a complete graph (i.e. they are fully connected). Although full Boltzmann machines have a higher number of parameters with respect to RBMs, they are not used in practice due to the high computational cost of training and, to date, the true potential of large-scale, full Boltzmann machines is not known.

The second direction for training RBMs is based on QA, a model of quantum computation that encodes problems in the energy function of an Ising model (QA was discussed in §8). Specifically, [137,138] make use of the spin configurations generated by a quantum annealer to draw Gibbs samples that can then be used to train an RBM. These types of physical implementations of RBMs present several challenges. Benedetti *et al.* [139] pointed out the difficulties in determining the effective temperature of the physical machine. To overcome this problem, they introduced an algorithm to estimate the effective temperature and benchmarked the performance of a physical device on some simple problems. A second critical analysis of quantum training of RBMs was conducted by Dumoulin *et al.* [132]. Here, the authors showed with numerical models how the limitations that the first-generation quantum machines are likely to have, in terms of noise, connectivity and parameter tuning, severely limit the applicability of quantum methods.

A hybrid approach between training ANNs and a fully quantum neural network is the quantum Boltzmann machine proposed by Amin *et al.* [140]. In this model, the standard RBM energy function gains a purely quantum term (i.e. off diagonal) that, according to the authors, allows a richer class of problems to be modelled (i.e. problems that would otherwise be hard to model classically such as quantum states). Whether these models can provide any advantage for classical tasks is unknown. Kieferova & Wiebe [141] suggest that quantum Boltzmann machines could provide advantages for tasks like reconstructing the density matrix of a quantum state from a set of measurements (this operation is known in the quantum information literature as *quantum state tomography*).

Although there is no consensus on the defining features of a quantum ANN, the last two decades have seen a variety of works that attempted to build networks whose elements and updating rules are based solely on the laws of quantum mechanics. The review by Schuld *et al.* [142] provides a critical overview of the different strategies employed to build a quantum ANN and highlights how most of the approaches do not meet the requirements of what can be reasonably defined as a quantum ANN. In particular, most of the papers surveyed by Schuld *et al.* failed to reproduce basic features of ANNs (for example, the attractor dynamics in Hopfield networks). On the other hand, it can be argued that the single greatest challenge to a quantum ANN is that the quantum mechanics is linear but ANNs require nonlinearities [143].

Recently, two similar proposals [144,145] have overcome the problem of modelling nonlinearities by using measurements and introducing a number of overhead qubits in the input and output of each node of the network. However, these models still lack some important features of a fully quantum ANN. For example, the model parameters remain classical, and it is not possible to prove that the models can converge with a polynomial number of iterations. The authors of the papers acknowledge that, in their present forms, the most likely applications of these models appear to be learning quantum objects rather than enhancing the learning of classical data. Finally, we note that, to date, there are no attempts to model nonlinearities directly on the amplitudes.

## 10. Learning with noise

Noise can play different, potentially beneficial roles in learning problems. In a classical setting, it has been shown that noise can alleviate two of the most common model-fitting issues: local optima and generalization performance. Perturbing gradients can help with the former by ‘jumping out’ of local optima, whereas perturbing training inputs or outputs can improve the latter.

The possibility of exploiting advantageously the effects of noise is particularly interesting in the context of quantum computation. Early quantum computers are expected to have too few qubits to implement full error correction and the community is actively looking for problems where noise not only does not destroy the computation but can play a beneficial role.

The analysis of noisy learning problems from a quantum perspective becomes particularly promising in selected cases. As we will discuss in this section, quantum resources allow efficiently noisy learning problems to be solved that would be otherwise classically hard. Although few results are known in this area, further research in this direction might provide new cases of a separation between the classical and quantum case in a learning setting.

The goal of this section is to inspire future research aimed at understanding how quantum learners behave in noisy settings. We begin by reviewing for quantum scientists a number of classical problems in ML that benefit from noise. We proceed with a brief introduction to standard ways of modelling errors in quantum computing aimed at ML practitioners. We conclude by discussing problems where quantum resources allow tasks to be performed that would be otherwise hard for a classical learner.

### (a) Classical learning can benefit from noise

#### (i) Noisy inputs

The first direct link between the addition of noise to the training inputs ${\left(x_{i}\right)}_{i=1}^{n}$ and Tikhonov regularization was drawn in [146]. Here, it is shown that optimizing a feed-forward neural network to minimize the squared error on noisy inputs is equivalent (up to the order of the noise variance) to minimizing the squared error with Tikhonov regularization on noiseless inputs.

Intuitively, this form of regularization forces the gradient of the neural network output *f*(*x*) with respect to the input *x* to be small, essentially constraining the learned function to vary slowly with *x*: neighbouring inputs are encouraged to have similar outputs.

An [147] also investigated the effects of adding noise to inputs, outputs, weights and weight updates in neural networks and observed that input (and sometimes weight) noise can, in some settings, improve generalization performance.

#### (ii) Noisy parameter updates

More recently, in [148], the addition of annealed i.i.d. Gaussian noise to the gradients has been empirically shown to help in optimizing complex neural network models. Indeed, stochasticity in the optimization process can also derive from evaluating gradients of the objective function with respect to randomly selected subsets of the training points (as in *stochastic gradient descent*). This can be intuitively compared to simulated annealing [149] because the natural variability in these ‘partial’ gradients can help local optima (and saddle points) to escape and the (decreasing) gradient step size can be directly compared to the annealing temperature.

The addition of noise to the update of model parameters was also adopted in [150]. There, as well as using random subsets of training points to evaluate gradients, at each iteration the parameter update is perturbed with Gaussian noise (with variance equal to the decreasing step size). After the initial stochastic optimization phase, it can be shown that this method, under specific conditions, will start generating samples from the posterior distribution over model parameters, allowing us to quantify model uncertainty and avoid overfitting at no extra computational cost.

#### (iii) Noisy outputs

In GP regression [24], on the other hand, noise in the training outputs ${\left(y_{i}\right)}_{i=1}^{n}$ helps avoid the inversion of an otherwise potentially ill-conditioned kernel covariance matrix *K*. Assuming additive isotropic Gaussian noise (with variance *σ*^2^), to evaluate model predictions, we only ever need to invert a matrix of the form *K*+*σ*^2^*I*. This can be practical as the kernel matrix is singular or ill-conditioned whenever training inputs are repeated or are very close in the Hilbert space associated with the kernel covariance function.

Finally, when training *generative adversarial networks* (GANs [151]), it has been shown that an overconfident ‘discriminator’ can hinder learning in the ‘generator’. In GANs in fact, a generative model (the ‘generator’) is trained by attempting to ‘deceive’ a ‘discriminator’ model into classifying the generated images as coming from the true data distribution. However, especially early on in training, there might be little overlap in the support of the data distribution and the generator. This can result in the discriminator predicting labels with very high confidence and, as well as potentially overfitting, in making the discrimination decision depend very weakly on the generator’s parameters. To address this issue, labels (i.e. true, fake) can be ‘fuzzied’. Specifically, for each training point, the discriminator will assume that all *K* labels have probability at least *ϵ*/*K* of occurring, with the true label having probability (1−*ϵ*)+*ϵ*/*K*. This corresponds to assuming that with probability *ϵ*/*K* labels are sampled at random and, indeed, labels can just be flipped randomly in practice. Effectively, this keeps the model from becoming too confident in its predictions by making it suboptimal to shift all the probability mass on the true label. This technique is called label smoothing [152] and it has been shown to help retain the training signal for the generator [153], as well as increase the robustness of classifiers to adversarial examples [154].

### (b) A classical/ quantum separation in learning under noise

To address learning under noise in a quantum setting, it is necessary to discuss what type of noise affects quantum computers. The works by Preskill [17] and Breuer & Petruccione [155] cover the topic extensively. A simple model of quantum errors, usually employed in numerical simulation of noisy quantum devices, makes use of a weighted combination of two kinds of error: bit flips and phase flips. We can justify this simple type of modelling because, in the most common error-correcting codes, errors are detected by projecting more complex errors into convex combinations of bit and phase flips. Given a quantum state *ψ*=*α*_0_*e*_0_+*α*_1_*e*_1_, a bit flip error turns the state into $\tilde{\psi }=\alpha_{0}e_{1}+\alpha_{1}e_{0}$. Similarly, a phase flip error changes the relative phase of a quantum state, i.e. the resulting state is $\tilde{\psi }=\alpha_{0}e_{0}-\alpha_{1}e_{1}$. More complex and realistic models of errors include amplitude damping, leakage to higher levels and loss.

Many authors have studied how noise affects the learnability of a function in the quantum setting. The already mentioned work by Bshouty & Jackson [31] showed that DNF formulae can be efficiently learned under the uniform distribution using a quantum example oracle. This contrasts with the classical case (although proved in the statistical query model of learning) where Blum *et al*. [156] showed that DNFs are not learnable under noise with respect to the uniform distribution.

Another result that points to a separation between classical and quantum for a noisy learning problem has been recently proved by Cross *et al.* [157]. In this case, the learnability of parity functions under noise is discussed. It is widely believed that learning parity function under noise is not classically efficient [158] and the best classical algorithm is run in subexponential, but superpolynomial, time. Furthermore, the problem is an average-case version of the NP-hard problem of decoding a linear code [159], which is also known to be hard to approximate [160]. Both the classical and quantum problem are easy without noise. In [157], Cross *et al.* showed that in the quantum PAC model parity functions can be learned efficiently under the uniform distribution (with logarithmic overhead over the noiseless runtime). Their results have been generalized to linear functions and to more complex error models by Grilo & Kerenidis [161].

To summarize, in this section, we surveyed a number of classical results showing that noise in the inputs, outputs or in the parameters can have positive effects on learning algorithms. It would be interesting to investigate whether the type of noise encountered in quantum systems has a similar distribution and structure to the one commonly encountered in classical settings. In this case, ML algorithms would become ideally suited to run on non-fault-tolerant quantum hardware. Finally, further research is needed to identify new, noisy problems that only a learner equipped with quantum resources can solve.

## 11. Computationally hard problems in machine learning

Algorithms whose runtime is upper-bounded by a polynomial function of *N* are said to be *efficient*. Problems for which there exists an efficient algorithm are *easy*. Conversely, *hard* problems are those where no polynomial algorithm is known. An important class of easy problems is called P. The class of problems that are efficiently solvable by a quantum computer includes some problems that are not known to be in P.

The quantum algorithms surveyed in this review speed up efficient classical algorithms. Two types of speed-ups are obtained: polynomial or exponential. Polynomial speed-ups, although important from a practical point of view, do not prove that quantum computers are able to turn hard learning problems into easy ones. On the other hand, exponential speed-ups of algorithms that are already efficient face important challenges. Indeed, as we have seen for the matrix inversion algorithm discussed in §6, quantum algorithms for the analysis of classical data running in logarithmic time require an equally fast access to the memory. This can be obtained using a QRAM that, however, presents a number of issues (see §5).

To achieve an exponential speed-up despite the computational costs arising from accessing the memory, we are restricted to hard algorithms. This is because, for these algorithms, the polynomial time construction of the quantum state that encodes the dataset does not dominate over the speed-up. We discussed an example with such a property: the learnability of DNF formulae (§4). Classically, the best algorithm for learning DNFs runs in superpolynomial time. With quantum resources we can learn the same problem polynomially. Although these types of learning problems have limited practical applications, they suggest that an exponential separation between classical and quantum models of learning might hold in real-world problems.

In this section, we present a number of problems in ML that are believed to be computationally hard and are receiving considerable interest in the classical community. We do not expect that these problems, some of which are NP-hard, can be solved efficiently with a quantum computer. Recall that NP-hard is a class of problems for which there is strong evidence of a separation with P [162]. Our hope is to spark interest in the search for hard problems in ML with the kind of structure (see §2) that can be exploited by quantum computers. We also decided to include problems that are not hard in the computational complexity sense but whose high-degree polynomial runtimes make them intractable. For these cases, where slow (i.e. polynomial) memory access times can still be tolerable, even polynomial speed-ups might be of great practical relevance.

### (a) Tensor factorization

As modern datasets grow not only in terms of sheer dimension but also in the complexity of the structures required to store such data (e.g. multi-modal data, social networks, recommender systems and relational data [163–165]), it becomes ever more critical to devise methods able to distil concise and interpretable representations of this information. Tensor models offer a powerful way to address these learning problems. For instance, tensors naturally generalize the concept of the adjacency matrix for multi-relational graphs [166]. However, given the intrinsic multi-dimensional nature of these objects, tensor-based learning problems are typically computationally hard and require large amounts of memory; therefore, they become quickly impractical in most applications. To this end, finding low-rank approximations of tensors (or more generally multi-linear operators), a natural generalization of the problem of matrix factorization (see [167] and references therein) has recently received significant attention from the fields of ML, inverse problems and compressive sensing. However, while for the matrix case the problem is amenable to efficient computations, moving to higher orders becomes significantly challenging. Indeed, in contrast to its matrix counterpart, low-rank tensor factorization, even when relaxed to a nuclear norm-regularized optimization problem, has been recently shown to be NP-hard [168]. Approaches have attempted to circumvent these issues by considering further relaxation of the factorization problem [169–174], but to this day a standard solution has yet to be proposed.

### (b) Submodular problems

Recently, several ML problems have been addressed via submodular optimization. Examples of such applications are very diverse, such as document summarization [175], social networks [176] or clustering [177] to name but a few. Submodularity characterizes a family of discrete optimization problems, typically entailing cost functions on sets in which the target functional exhibits a structure akin to that of convexity (or rather concavity) for continuous functions. We refer to Bach [178] for an in-depth introduction on the topic. For many submodular problems, it is possible to identify a corresponding convex problem via the so-called *Lovàsz* extension [179]. As a consequence, such problems can be solved using convex optimization methods, leading to efficient learning algorithms. However, for a wide range of these problems, the corresponding computational complexity, albeit polynomial, is of high order (e.g. $O(n^{5})$ with respect to the number *n* of the parameters; see for instance [180–182]), making them remarkably slow in practice. In this sense, an exponential (or even polynomial) decrease in the number of computations to solve a submodular problem, analogous to the one observed for fast linear algebra using quantum algorithms, could be the key to tackling practical applications.

### (c) Inference in graphical models

Probabilistic models in ML can be encoded in graphs. Graphical models of particular use are Bayesian networks [183] and Markov random fields [184]: directed acyclic and undirected graphs, respectively, where nodes represent random variables and edges denote dependence between variables. Operations like marginalization and conditioning can be performed by algorithms taking into account the specific connectivity of the given graph (i.e. message passing). While this offers a general framework for inference (i.e. evaluating the distribution of latent variables conditioned on observed ones), it has been shown, by reduction to Boolean satisfiability [185], that exact inference in these networks is NP-hard and that evaluating the normalizing constant *Z* (or partition function) for the joint distribution is in #P (a family of hard counting problems).

## 12. Conclusion and outlook

In this review, we surveyed a number of different quantum methods to tackle learning problems. Despite a number of promising results, the theoretical evidence presented in the current literature does not yet allow us to conclude that quantum techniques can obtain an exponential advantage in a realistic learning setting. Even in the case of quantum algorithms for linear algebra, where rigorous guarantees are already available, issues related to data access and restrictions on the types of problems that can be solved might hinder their performance in practice. In fact, near-future advances in quantum hardware development will be important to empirically assess the true potential of these techniques. In this regard, we note how the great majority of the QML literature has been developed within the quantum community. We believe that further advances in the field will only come after significant interactions between the two communities. For this reason, we tried to structure this review to present the different topics in a way that is familiar to both quantum scientists and ML researchers. To achieve this goal, we put great emphasis on the computational aspects of ML. Although this perspective has the obvious advantage of providing an agile way for discussing quantum algorithms (that mostly focus on accelerating the runtime with respect to their classical counterparts), the reader should keep in mind that statistical problems (like determining the generalization performance of an algorithm) are equally relevant. The approach taken in this review has also left some interesting papers aside (e.g. [186]). We invite the reader to consult [7,8,187] for a review that includes these works.

In §3, we discussed how the computational cost represents one of the major challenges for the future of ML. In particular, polynomial scaling in the number of data points might not be adequate in the age of large-scale ML. The quantum algorithms presented here allow the complexity of some, currently used, regularization methods to be reduced. We classified the quantum approaches into four main categories: linear algebra, neural networks, sampling and optimization. The QML algorithms based on linear algebra subroutines are those that promise the greatest computational advantages (i.e. exponential). However, it is not clear whether fundamental limitations related to how quickly these algorithms need to access the memory might compromise their ability to speed up the analysis of classical data. Quantum methods for training neural networks, for sampling and for optimization, provide so far mostly quadratic advantages and some of these might be implementable on first-generation quantum computers. Unfortunately, the theoretical framework on which they are based is not yet well established (e.g. the quantum Boltzmann machines described in §9) and only practical experiments will determine their true performance.

To summarize, the works surveyed in this review, including the theoretical evidence presented in §4, suggest the possibility of a quantum speed-up for some ML problems. However, the extent of these speed-ups, and consequently the impact of these methods on practical problems, remains an open question.

We identified a number of promising directions for the field. First, exploring the trade-offs between noise, generalization performance and hardness in a quantum context (§10). This is particularly interesting for first-generation quantum hardware that most likely will not be fault-tolerant. Second, deepening our understanding of how quantum resources can affect sample and time complexity, even for problems that are already known to be efficient. Significant work has already been done but some areas like statistical learning theory are yet to receive a thorough analysis in a quantum context. Third, determining whether a QRAM of the size required to handle large datasets can be constructed on a physical device (§5). Fourth, understanding whether there exist non-polynomial problems in ML that can be tackled efficiently using quantum resources (§11). This direction is arguably the most relevant for finding quantum algorithms capable of demonstrating an uncontroversial speed-up in a learning context, and this is indeed the general quest of quantum computation.

## Data accessibility

This paper has no additional data.
